# Current Role and Future Perspectives of Cardiac Rehabilitation in Heart Disease

**DOI:** 10.31083/j.rcm2503076

**Published:** 2024-02-27

**Authors:** Lamei Yang, Yi Bai, Li Li, Sisi Zheng, Xiaoli Yan, Li Yu, Shilan Luo

**Affiliations:** ^1^Department of Medical Record Management, West China Second University Hospital, 610041 Chengdu, Sichuan, China; ^2^Key Laboratory of Birth Defects and Related Diseases of Women and Children (Sichuan University), Ministry of Education, 610041 Chengdu, Sichuan, China; ^3^Health Management Center, The First Affiliated Hospital of Chongqing Medical University, 400016 Chongqing, China; ^4^Department of Geriatric Cardiology, The Second Affiliated Hospital of Chongqing Medical University, 400010 Chongqing, China

**Keywords:** cardiac rehabilitation, heart disease, secondary prevention, model of delivery

## Abstract

As a comprehensive secondary prevention program, cardiac rehabilitation (CR) is a beneficial and cost-effective intervention for patients with heart disease, but the participation rate of patients in CR is low globally. In recent years, due to the COVID-19 pandemic and scientific and technological advances, an increasing number of alternative CR modes have been developed, such as remote CR, home-based CR, hybrid CR and virtual CR. These alternative CR modes represent changes and new opportunities for patients with heart disease. In this review, we will discuss in detail the impact of CR on patients with different types of heart disease, review the various alternative CR models, and explore some prospects for the future of CR in the field of heart disease.

## 1. Introduction: Cardiovascular Disease

The World Health Organization (WHO) and various national medical associations called for greater attention to cardiovascular disease, and relevant studies have shown that the prevalence of cardiovascular disease decreased in some areas, especially in higher income countries. However, cardiovascular disease (CVD) remains one of the leading causes of death worldwide [1]. The WHO reported that CVD kills 17 million people per year, accounting for approximately 31% of all deaths worldwide; this number is expected to rise to more than 23 million by 2030 [2]. With advances in disease screening technology, acute treatment methods, and control of related risk factors, most patients with CVDs experience prolonged survival. Because patients suffer from cardiovascular disease, the probability of death and complications is further increased compared with healthy people, and CVDs continue to impose substantial medical and economic burdens on society [3, 4]. Improving the quality of life of cardiovascular patients and reducing complications and mortality is a key focus of medical personnel, medical managers and policy-makers. In this context, cardiac rehabilitation (CR) was proposed in the 1960s; it was originally used to guide middle-aged men to exercise after acute myocardial infarction. With advances in medical technology, evidence of the benefits of CR has accumulated in the past few decades. Current clinical guidelines strongly recommend that cardiovascular patients participate in CR [5]. In this article, we will focus on the heart disease population and explore the current role of CR and the vision for the future in the heart disease population.

## 2. What is CR?

CR is a guideline-recommended secondary prevention program involving a multidisciplinary team that includes exercise training, medical management, patient education, and psychological and nutritional interventions, including weight management, blood pressure control, diabetes and lipid management, and smoking cessation, to help patients with CVD improve their health and prevent complications [6]. Many studies have proven that CR can reduce the risk factors for CVD, mortality, readmission rate, and health care costs; improve cardiorespiratory health; and improve the survival rate and quality of life of cardiovascular patients. In the United States, researchers have calculated that if the CR participation rate is increased from its current level (20%) to 70%, approximately 180,000 fewer hospitalizations would occur each year, and 25,000 lives could be saved [7]. Therefore, CR as a secondary prevention is likely to become the focus of cardiovascular patient management [8, 9].

**Fig. 1.**
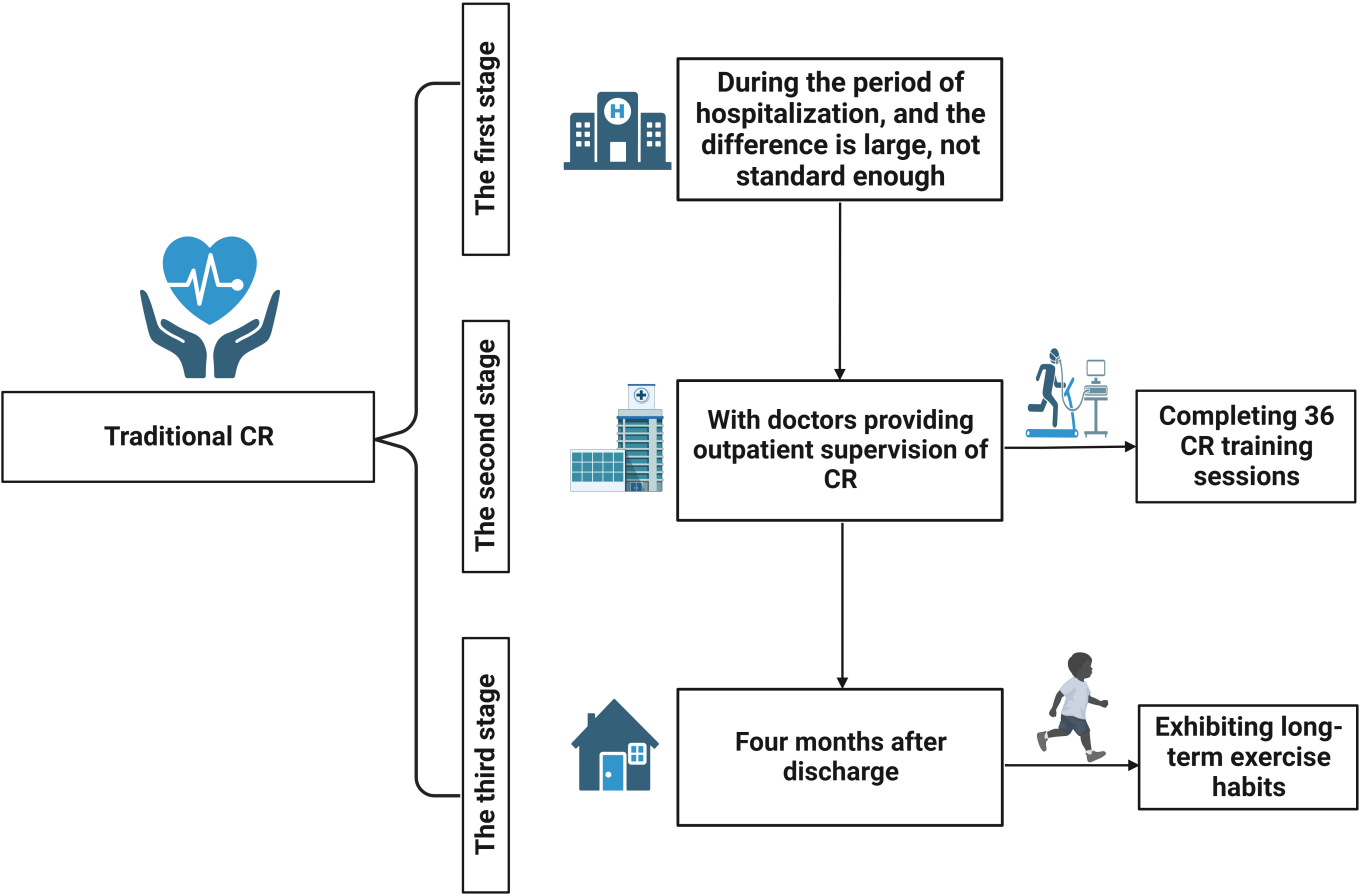
**Traditional cardiac rehabilitation process**. CR, cardiac rehabilitation.

Traditional CR is divided into three stages (Fig. 1). The first stage is CR under the guidance of doctors during hospitalization. Due to reductions in the length of hospital stay, this first stage of CR varies greatly and lacks sufficient standardization. The second stage of CR occurs in the four months after hospital discharge, with doctors providing outpatient supervision of CR, in which cardiovascular patients complete 36 CR training sessions. The third stage starts four months after discharge, at which time participants may exhibit long-term exercise habits. Studies have shown that only approximately a quarter of patients currently participate in the third stage of CR, and participation is influenced by gender, ethnicity, socioeconomic status, and geographic location [10, 11].

With the end of the global COVID-19 pandemic, social interaction is resuming, and CR methods have exhibited a large number of practical opportunities [12]. In this article, we discuss the current role and future prospects of CR in heart disease patients with the aim of providing a summary of the field and new ideas for future CR researchers.

## 3. The Benefits of Exercise Training for Heart Disease Patients

“Heart disease” includes all diseases of the coronary arteries, the electrical system of the heart, and the mechanical function of the heart. Exercise-based CR can provide benefits for almost all types of heart disease. The American Heart Association and the American College of Cardiology strongly recommend CR. Exercise in CR undoubtedly provides many benefits for cardiovascular patients. A meta-analysis of 23,430 adult patients with myocardial infarction, angina, or percutaneous coronary intervention (PCI) included 85 randomized controlled trial (RCTs) demonstrated that exercise-based CR reduces cardiovascular mortality, the incidence of cardiac events, and hospitalization and that there is exceptional evidence for the cost-effectiveness of improving health-related quality of life through CR [13]. In addition, a meta-analysis of 5783 patients with heart failure that included 44 RCTs showed that short-term participation in CR (less than 1 year) had no effect on the mortality of patients with heart failure but reduced the overall hospitalization rate, while long-term participation in CR (more than 1 year) could reduce the mortality of patients with heart failure. CR may reduce hospitalizations due to heart failure and has been shown to improve quality of life according to any measure of health-related quality of life [14]. One meta-analysis of 924 patients with congenital heart disease that included 15 RCTs demonstrated small improvements in peak cardiorespiratory fitness (CRF) and mean daily physical activity (PA). There was insufficient evidence of improvement in health-related quality of life [15]. Another meta-analysis of 404 heart transplant patients from 11 studies showed that exercise significantly improved the functioning of heart transplant patients in the short term and that high-intensity interval training provided greater benefits than moderate-intensity continuous training [16]. A study in a developing country of 6380 patients with heart disease from eight countries, including China, Bangladesh, and Brazil, demonstrated increased exercise capacity after participating in exercise-based CR (mean increase in peak oxygen uptake of 3.13 mL/kg/min, 95% CI: 2.61–3.65 mL/kg/min) [17].

Exercise plays a key role in CR. Various studies have shown that physical activity during CR reduces overall mortality, cardiovascular mortality, complication rates, readmission rates, and health care costs while improving patients’ quality of life [13]. As the core aspect of CR, exercise can increase the physical endurance of patients with heart disease, reduce blood lipids and blood pressure, and improve peak oxygen consumption [18]. Exercise also influences the cardiovascular system of patients with heart disease and, to a certain extent, can slow the progression of coronary atherosclerosis [19]. Furthermore, exercise also has beneficial effects on the lungs and skeletal muscles of patients and provides a non-negligible improvement in overall body function [20, 21]. There is further evidence that changes in lifestyle and risk factors are no less effective in reducing CVD mortality than treatment techniques, so in the future, the importance of CR should not be ignored [22].

## 4. The Current Role of CR for Heart Disease Patients

Many studies have confirmed that exercise-based CR can provide many benefits for cardiovascular patients, but the specific impact of CR on different types of heart disease remains unclear. Below, we summarize several common types of heart disease.

### 4.1 Coronary Heart Disease

Coronary heart disease (CHD) involves the formation of atherosclerotic plaques in one or more coronary arteries, which can lead to the complete closure of the blood vessels, leading to myocardial infarction or narrowing of the blood vessels, reducing the blood supply to the heart muscle and causing angina pectoris. CHD is the most common cause of referral to CR worldwide [22]. Improving quality of life is particularly important as an increasing number of people with CHD survive for many years, and CR is recommended as a Class I secondary prevention option in international guidelines [23].

A meta-analysis including 51 studies and 10,286 patients with CHD showed that CR can significantly reduce the levels of low-density lipoprotein cholesterol, triglycerides and total cholesterol, increase the levels of high-density lipoprotein cholesterol [24], and improve endothelial function. In addition, fasting blood glucose, blood pressure and inflammatory markers such as C-reactive protein (CRP) in CHD patients were improved [18]. There is evidence that participation in CR for at least 6–12 months can slow the progression of atherosclerosis and further limit myocardial remodeling [25].

Although the benefit of CR for patients with CHD has been confirmed in various studies, it has been questioned due to uncertainty regarding mortality, lack of health-related quality of life data, the inclusion of RCTs only containing low-risk patients, absence of data from low- and middle-income countries, and lack of trials including modern CHD treatment. Therefore, a 2023 meta-analysis of an updated Cochrane review included 23,430 patients from 85 RCT studies who were followed for ≥6 months, with a median follow-up of 12 months; studies were identified through database searches for publication dates from June 2014 to September 2020. The study subjects were adult patients with myocardial infarction, PCI, coronary artery bypass grafting or angina pectoris who participated in CR, and the results showed that cardiovascular mortality [risk ratio (RR): 0.74, 95% confidence interval (CI): 0.64–0.86, number needed to treat (NNT): 37], the hospitalization rate (RR: 0.77, 95% CI: 0.67–0.89, NNT: 37) and the incidence of myocardial infarction (RR: 0.82, 95% CI: 0.70–0.96, NNT: 100) were significantly lower and health-related quality of life was improved [13]. However, the overall mortality (RR: 0.96, 95% CI: 0.89–1.04) and that of coronary artery bypass grafting patients (RR: 0.96, 95% CI: 0.80–1.15) was significantly lower, and percutaneous coronary intervention (RR: 0.84, 95% CI: 0.69–1.02) did not have a significant effect [13]. CR can provide many benefits for patients with CHD, and this updated meta-analysis had wide coverage and included representative samples from 21 trials involving 7851 patients in low- and middle-income countries.

In conclusion, for patients with CHD, CR can reduce cardiovascular mortality, the hospitalization rate and the incidence of cardiovascular events, improve quality of life, reduce blood lipemia, blood pressure and blood sugar, and improve body function and heart function. However, in this meta-analysis [13], the subgroup sensitivity analysis of 16 studies reporting cardiovascular mortality and all-cause mortality revealed higher overall mortality (RR: 0.85, 95% CI: 0.74–0.98) and cardiovascular mortality (RR: 0.79, 95% CI: 0.68–0.92). This result is contrary to the results presented in this meta-analysis. Therefore, more studies are needed to determine whether CR can improve the all-cause mortality of patients with CHD.

### 4.2 Heart Failure

Heart failure (HF) means that due to the systolic function and/or diastolic function of the heart, the amount of venous blood cannot be fully discharged from the heart, resulting in blood stasis of the venous system and insufficient blood perfusion of the arterial system, resulting in cardiac circulation disorder, leading to pulmonary congestion and vena cava congestion [26]. HF is the end stage of various CVDs. Patients with HF often have poor quality of life, a high risk of hospitalization, high medical expenses and a poor survival rate. In a previous study, the 1-year survival rate was 81.3% (95% CI: 89.9–81.6), the 5-year survival rate was 51.5% (95% CI: 51.0–52.0), and the 10-year survival rate was 29.5 (95% CI: 28.9–30.2) [27]. An increasing number of systematic reviews and meta-analyses have demonstrated that CR can benefit patients with HF [14, 28].

A 2019 Cochrane review, which was updated in 2023, showed that patients with HF who participated in CR had lower rates of all-cause hospitalization and HF hospitalizations, as well as improved health-related quality of life [14, 29]. In the end, the review included 8728 patients from 60 RCTs, and the subjects were patients with HF with a mean age of 63 years, a low ejection fraction (mean ejection fraction of 32%), and a New York Heart Association heart function score of grade II or III. All-cause mortality was assessed after a median follow-up period of 6 months [RR: 0.93, 95% CI: 0.71–1.21], along with the all-cause hospitalization rate [RR: 0.69, 95% CI: 0.56–0.86], hospitalization rate for HF [RR: 0.80, 95% CI: 0.70–1.06], and the total Minnesota Living with Heart Failure Questionnaire (MLWHF) score [mean: –7.4, 95% CI: –10.3 to –4.5]. This latest Cochrane review confirmed that participation in CR can reduce the risk of all-cause hospitalization and heart failure hospitalization by 25% to 30% in patients with heart failure and that an MLWHF score ≥5 is clinically significant, indicating that health-related quality of life in patients with heart failure improved after CR. Therefore, although the reduction in overall mortality in patients with heart failure was not very significant, participation in CR reduced all-cause hospitalization and heart failure hospitalization while improving health-related quality of life and reducing healthcare costs [29].

### 4.3 Heart Transplantation

Heart transplantation (HT) is the end-stage choice for patients with chronic heart failure, and the survival rate, functional status and quality of life of patients are effectively improved after heart transplantation [30, 31]. The International Society of Heart-Lung Transplantation recommends CR before and after heart transplant surgery, noting that CR for patients waiting for a heart transplant can reduce readmission rates, reduce mortality and improve prognosis after a heart transplant; the society suggests that routine CR after heart transplant can improve exercise capacity, endothelial function, and skeletal muscle function and reduce cardiovascular risk factors [32].

Studies on patients who participated in CR 3–4 days per week, including aerobic exercise and resistance training, prior to HT have assessed patients’ quality of life (using the SF-36) and physical activity levels (using the International Physical Activity Questionnaire (IPAQ)), showing that patients who participated in CR have an average increase in physical activity levels of 32% three months after transplantation. Quality of life improved by an average of 43%; however, the study did not include a control group and was only one report on three patients. Nevertheless, it did confirm the feasibility and safety of CR participation before HT [33].

A 2017 Cochrane review of 10 RCTs including 300 heart transplant patients followed for a median of 12 weeks showed that compared with a control group that did not exercise, the group that engaged in exercise-based CR exhibited increased exercise capacity (VO2peak MD (mean difference): 2.49 mL/kg/min, 95% CI: 1.63 to 3.36, N = 284, studies = 9, moderate quality evidence). High-intensity interval training improved exercise performance to a greater extent than sustained moderate-intensity exercise (MD: 2.30 mL/kg/min, 95% CI: 0.59 to 4.01, N = 16, 1 study). There was no difference in health-related quality of life between the CR group and the control group [34].

In short, CR can improve the exercise ability of patients after HT [16, 35]. However, the number of available studies was limited, and more studies are needed in the future to demonstrate the effectiveness of CR for reducing mortality and other aspects in HT patients.

### 4.4 Valvular Heart Disease

Valvular heart disease refers to a heart disease caused by stenosis or incomplete closure of the heart valve. This is typically an age-related degenerative disease, mainly affecting individuals aged 50 years and older. There are few studies on the benefits of CR participation in these heart disease patients, but CR is still recommended for valvular heart disease based on clinical experience and expert opinion [36].

One study of patients who participated in CR after heart valve surgery assessed their peak oxygen uptake (VO2 peak) or performance on the 6-minute walk test (6MWT) after completing CR and found a 16% improvement in VO2 (*p* < 0.0001) and a 13% improvement in the 6MWT distance in the 146 patients who completed CR [37]. Additionally, the clinical incidence of nonparticipation in CR was higher, with an adjusted hazard ratio of 2.46 (95% CI: 1.26–4.80). Therefore, CR after heart valve surgery improved exercise capacity and reduced morbidity, but older adults and minorities were less likely to participate in or complete CR [37]. A 2021 Cochrane review included six RCTs of 364 patients who underwent open or percutaneous heart valve surgery, but the evidence quality was so low that the authors were unable to draw conclusions about its impact on mortality, hospitalization or health-related quality of life. CR increased peak oxygen uptake across all measurements (mean difference 2.38 mL/kg/min, 95% CI: 0.36–4.40 mL/kg/min, five trials, 294 patients, medium-quality evidence) [38].

Thus, CR was found to increase exercise capacity in patients with valvular heart disease, but more research is needed to support its other benefits.

### 4.5 Congenital Heart Disease

Congenital heart disease refers to the presence of a series of structural abnormalities in the heart at birth, and approximately 1% of babies worldwide are born with congenital heart disease [39]. With advances in medical technology, people with congenital heart disease are living longer. Due to the decreased cardiopulmonary function and poor exercise ability of patients with congenital heart disease, they have lower quality of life; however, CR has been demonstrated to have beneficial effects for patients with congenital heart disease [40, 41].

A meta-analysis of eight controlled trials in children and adolescents showed that exercise had a positive effect on peak oxygen consumption [42]. A Cochrane review of 15 RCTs including 924 patients, including 5 RCTs in children and adolescents (n = 500), and 10 RCTs including 424 adult patients showed a small increase in cardiorespiratory fitness compared to that of controls. The mean difference was 1.89 mL/kg/min (95% CI: –0.22 to 3.99, n = 732, moderate certainty evidence), and there was an increase in muscle strength (MD: 17.13, 95% CI: 3.45 to 30.81, n = 18, moderate-certainty evidence) [15].

Another study evaluated metabolic equivalent task units, exercise time, and VO2 Max before and after CR and showed a 1.3 increase in metabolic equivalent task units (95% CI: 0.7–1.9; baseline mean, 8.1), an exercise time increase of 66.4 seconds (95% CI: 21.4–111.4 seconds; baseline mean: 536.1 seconds), and a oxygen uptake increase of 2.5 mL/kg/min (95% CI: 0.7–4.2 mL/kg/min; baseline mean: 20.2 mL/kg/min), demonstrating improved exercise capacity in patients with congenital heart disease who participated in CR [43].

Relevant studies have confirmed that both children and adults with congenital heart disease exhibit improved cardiorespiratory fitness and exercise ability after CR, but there is insufficient evidence of improvements in mortality, readmission rates and quality of life, and more relevant studies are needed in the future.

### 4.6 Atrial Fibrillation

Atrial fibrillation (AF) is a common arrhythmia. After its occurrence, it can easily increase breathing difficulties and movement difficulties as well as the risk of clinical events, especially stroke and heart failure, which represent considerable hidden dangers to health [44]. In a 2017 Cochrane review of 6 RCTs including 421 patients with different types of AF, CR did not lead to significant differences in mortality, adverse cardiovascular events, or quality of life in patients with AF. Two pieces of moderate-quality evidence showed that AF patients who participated in CR had improved exercise capacity, with peak measures after CR averaging 3.76 higher than those of controls (95% CI: 1.37–6.15) [45].

At present, there are few studies on the impact of CR on patients with AF, and evidence indicating the true impact of CR on the mortality and incidence of cardiovascular events in patients with AF is lacking. In the future, high-quality RCTs are needed to study the impact of CR in patients with AF.

## 5. Current Status of CR Implementation

Unfortunately, only approximately half of countries currently provide CR, and CR programs are virtually non-existent in low- and middle-income countries, which have the highest and most rapidly increasing prevalence of CVD [46, 47]. Only about a quarter of cardiovascular patients who participate in CR do so, and participants vary by gender, race, socioeconomic status, and geographic location of center-based CR (CBCR) [10]. Because CR is not covered by health insurance in most countries and the benefits of CR are greatly underestimated by clinicians, CR is underutilized, and the lack of an evidence-based, recommended standard of use of CR also contributes to the uneven distribution of participation rates.

The latest data from the 2019 National Audit of CR in the UK showed that of 135,861 patients diagnosed with CHD, 68,074 received CR, a participation rate of approximately 50.1%. Due to the impact of the COVID-19 pandemic, the number of hospitalizations for heart disease has decreased by 40%, and the number of people participating in CR has decreased correspondingly [48, 49, 50]. In the United States, data from some CR registries indicate that only approximately one-third of patients participate in CR after a heart attack, and those who are female, Black, or uneducated are less likely to participate in CR [51]. In a study that evaluated CR referrals and participation at 131 hospitals in 27 countries in Europe, approximately 46% of patients received referrals to CR centers, and 69% of patients referred to CR centers attended more than half of the CR classes [52]. In developing countries, such as China, only 30 hospitals (24%) out of 124 Tier 3 hospitals provide CR services, or only 2.2 hospitals for an average population of 100 million [53].

There are many reasons for the low CR participation and completion rates in patients with heart disease. For example, there are differences among hospitals in the referral of patients to CR [54]. It is very important for medical staff to encourage patients to participate in CR, but most clinicians do not pay attention to CR referrals [55]. An investigation showed that clinicians lack an understanding of the benefits of CR and do not know how to make referrals [56]. CR programs are lengthy, which also leads to low patient completion rates. Although some high-income countries, such as the United States, have developed a reimbursement policy for CR, countries around the world still lack a consistent reimbursement strategy, and CR centers are often far from participants, which may explain the low participation rates [46, 57, 58]. Among the populations with low participation rates, rural, remote mountain-dwelling and ethnic minority individuals also have fewer CR opportunities, mainly due to a lack of awareness of the benefits of CR and a lack of support from CR professionals [59].

## 6. Future Perspectives for CR for Heart Disease Patients

CR is a highly beneficial form of rehabilitation for heart disease patients. To increase the participation rate of CR and allow more patients to benefit from it, countries have developed corresponding countermeasures. For example, the United States has set a goal of increasing CR participation to 70% by 2022, with the goal of saving 25,000 lives and preventing 180,000 hospitalizations per year [7]. The UK plans to increase the national CR participation rate from the current level (50%) to 85% by 2028 [60].

Due to the generally low CR participation rate and the COVID-19 pandemic, which led to the closure or suspension of activity at most CR centers around the world. In China, social distancing was only fully relaxed in December 2022. Due to the large challenges of administering traditional CBCR, such as the pandemic, long distances to centers, and lengthy periods of intervention, the participation rate of CBCR is low. These results have also led to increased demand for more alternative models. A lot of virtual and remote CR have been implemented everywhere. While it is still unclear exactly how virtual and remote CR should be implemented and what impact it will have across different populations, these are alternative models of CR that should be encouraged, and more importantly, a greater diversity of methods to participate in CR is expected to increase CR participation rates in the future [61, 62]. At the same time, an increasing number of researchers have explored remote CR, home-based CR (HBCR), and a combination of HBCR and CBCR, combined with information technology, including the installation of wearable devices and the development of artificial intelligence-based CR assistance systems. Such methods may provide patients with remote and personalized interventions, use information technology to track indicators, remind and encourage patients to complete the entire rehabilitation program remotely, and use information technology to carry out virtual reality CR training [63, 64]. Scientific and technological intervention refers to the integration of information technology into the care of patients participating in CR through the internet, digital and telephone monitoring and evaluation. Research has shown that this new mode of delivering CR assisted by advances in science and technology has comparable effects as CBCR, can improve exercise ability, quality of life, physical function, reduce anxiety and depression, improve medication adherence, improve risk factors, and reduce heart-related hospitalizations [65, 66].

**Fig. 2.**
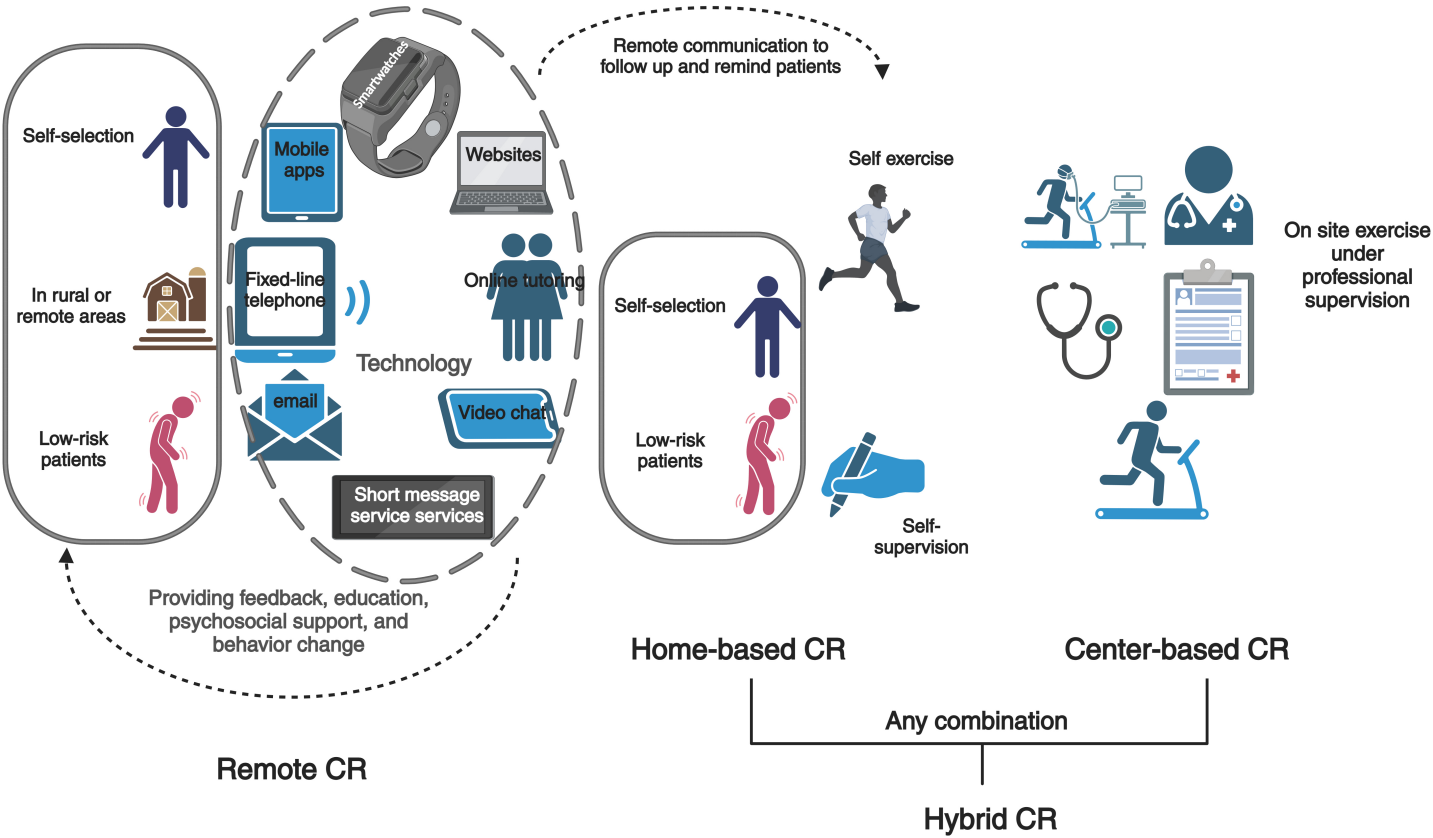
**Multiple alternative models of cardiac rehabilitation**. CR, cardiac rehabilitation.

Remote CR is often an alternative mode chosen by low-risk patients, who live far from the CBCR, or for other reasons (Fig. 2). In one study [67], patients who participated in remote CR were given a digital CD guide created by CR center staff (which included explanations of heart failure, warm-up exercises, aerobic exercise, and the possible need for emergency medical attention), and were given biweekly telephone counseling by a CR specialist for five months after discharge. The results showed that the emergency readmission rate was lower than that of the non-CR group [67]. In another study [68], patients who participated in remote CR were given a web application, with weekly live video consultations to assess vital signs and receive feedback. In the end, patients’ satisfaction with remote CR was generally high [68]. In short, the use of information and communication technology is the family program for remote CR. Participants can be provided with feedback, education, psychosocial support and behavior changes through mobile apps, smartwatches, fixed-line telephone, short message service services, email, websites, online tutoring and video chat [69]. Future research should make more use of technology to achieve a more comprehensive, interactive and personalized remote CR, and explore the sustainability of remote CR effects. Additionally, studies have shown that for low-risk cardiac patients, there is no significant difference in the safety and efficacy between remote CR and CBCR [61, 62].

The American Heart Association (AHA), American Association of Cardiovascular and Pulmonary Rehabilitation (AACVPR), and American College of Cardiology (ACC) have issued and approved HBCR [61]. HBCR is more of an alternative option for low-risk patients. The difference is that medical personnel cannot provide face-to-face guidance through remote communication for patient follow-up and reminders, because of the limitations of time and distance. Western countries have gradually implemented this method (Fig. 2). Relevant studies have found that HBCR can improve the participation rate of patients who are unable to participate in CBCR, and the treatment cost is also lower. Unfortunately, in the United States, CBCR reimbursement is generally supported and covered by Medicare, but HBCR is generally not covered by insurance reimbursement. The lack of financial compensation for HBCR is a barrier to the widespread implementation of HBCR in the United States, but some countries, such as Australia, the United Kingdom and Canada, have included HBCR reimbursement in their national health insurance policies [61]. In short, evidence shows that HBCR is similar to CBCR in terms of improvements in functional ability, health-related quality of life and risk factor control, and its compliance rate is better than that of CBCR. However, there is still a lack of research on the impact of HBCR on clinical events, and there is no comprehensive policy related to medical insurance reimbursement in many countries. Thus, more research is needed in the future to provide more empirical evidence of its safety for high-risk groups [61, 70].

HBCR and hybrid CR modes are recommended for low-risk patients who are unable to participate in a CBCR [61]. Hybrid CR refers to a combination of center- and home-based counseling and exercise, and is any combination of supervised center-based and supervised home-based exercises (Fig. 2) [71]. The hybrid CR model combines CR center and home CR, effectively increasing the accessibility and flexibility for patients to participate in CR. One study randomly assigned 270 patients after myocardial infarction to either a hybrid CR or a traditional CBCR [72]. The hybrid CR consisted of eight sessions of central exercise followed by eight weeks of home training, accompanied by a physiotherapist’s home visit every two months. The traditional CBCR program consisted of 40 center-based workouts. There was no significant difference in health-related quality of life between the two groups at 6 and 12 months, but direct medical costs were lower in the hybrid CR group [72]. Another study randomly divided 80 patients after myocardial infarction or cardiovascular surgery into hybrid CR and CBCR [73]. The hybrid CR began with exercise 3 times a week at the center, and after a month, patients began monitoring their heart rate at home, completing the exercise at home, with sessions at the center reduced to 2 times a week at weeks 6–11, once a week at weeks 11–17, and once every two weeks at weeks 18–25. The sessions lasted approximately one hour and focused on counselling, education and support. The control group underwent central CR three times a week for 24 weeks. The results showed that the hybrid CR group participated in more exercise sessions, cost was reduced by an average of 50%, and Body Mass Index (BMI) was reduced in the hybrid CR group (Hybrid CR group: 28.1 ± 4.9 kg/$m^{2}$ to 27.5 ± 4.8 kg/$m^{2}$; CBCR group: 28.4 ± 3.0 kg/$m^{2}$ to 27.9 ± 3.6 kg/$m^{2}$, *p* = 0.002). Project completion rates were 92% for the hybrid group and 76% for the central group [73]. Hybrid CR is designed to provide patients with exceptional options, and the primary purpose is to achieve the benefits of CR, while a single home-based or center-based exercise and counseling is unlikely to help patients produce the best outcomes and highest adherence. In the future, research could focus on patient-centered, personalized CR models, and make full use of technology, such as mobile phone apps and WeChat public accounts, to help patients increase exercise while changing risk factors.

Virtual reality refers to the use of technology to create a simulated environment. To create some tasks with therapeutic purposes for the situation, studies have used virtual reality to launch a “clinical arcade”, that is, combined virtual reality (VR) technology and rehabilitation, to achieve rehabilitation while playing; this approach may help individuals recover through recreational rehabilitation [74]. Studies have shown that virtual reality increased the motor ability of CR participants (pooled mean difference: 49.55, 95% CI: 30.59–68.52, *p* < 0.00001, moderate-certainty evidence) and improved quality of life and anxiety [62, 75]. Future studies can also be conducted in combination with virtual reality or apply virtual reality combined with HBCR or CBCR.

At present, the participation rate of CR is low worldwide, which is partly because clinicians have not paid attention to CR referral and education. Some studies have developed a scale to assess the attitude and support of medical staff toward CR, which can be used in future studies. At the same time, more studies are needed to examine the reasons behind the low participation rates [76]. Due to the lack of understanding of CR by some clinicians, medical personnel with stronger associations with CR should be included in the future to increase the human resources for CR. Additionally, it is hoped that clinicians can fulfil their duty of informing and including patients’ referrals to CR through the discharge orders (indicating the latest CR location and contact information for patients). Patients with low and medium risk levels can access HBCR programs through CR doctors, make short videos related to CR promotion and provide them to cardiovascular patients through mobile devices associated with cardiovascular patients and hospitals. In the future, if people participate in HBCR, tracking and follow-up through technology will increase data collection on its outcomes.

This paper effectively summarized the role of CR in cardiac patients, explained a variety of alternative models of CR, and provided a reference direction for future CR researchers. However, this study also has some limitations, such as the limitation of research methodology. Since the review is a secondary study, the quality of the included references directly affects the quality of this study. In short, CR represents a promising comprehensive secondary prevention method, and we hope that future research focuses on the following aspects: (1) the development of a low-cost, high-value CR model that can be combined with HBCR, virtual reality CR, community-based CR and remote CR; (2) use of artificial intelligence combined with other disciplines to develop personalized CR programs; (3) exploration of the reasons for the low participation rate, starting with the perspective and attitudes of medical staff to CR, and further development of the referral scheme to achieve a high participation rate; and (4) further RCTs to increase the evidence of CR’s effectiveness, especially for patients with atrial fibrillation, heart transplantation, congenital heart disease, and valvular heart disease.

## 7. Conclusions

CR is a guideline-recommended secondary prevention program involving a multidisciplinary team that helps patients with CVD improve their health and prevent complications. Although CR has different effects on different types of heart disease, many studies have confirmed its overall health benefits. However, the participation rate is generally low, and CR still faces many challenges.

In summary, future studies should consider how to achieve greater recruitment of patients with heart disease to participate in CR, and more studies are needed to promote the formulation of policies related to CR, especially regarding CR health education for patients, patients’ families and medical staff and the promotion of automated referral and medical reimbursement. Additionally, new models of CR that combine remote CR with science and technology should be explored, such as the impact of the diversity of HBCR on high-risk groups or (through multicenter studies) the impact of CBCR and virtual or remote CR. In the future, CR, as a very important secondary prevention intervention, will occupy a very important position in the field of cardiovascular treatment.

## Abbreviations

CR, cardiac rehabilitation; WHO, world health organization; CVD, cardiovascular disease; COVID-19, coronavirus disease 2019; RCT, randomized controlled trial; CRF, cardiorespiratory fitness; PA, physical activity; CHD, coronary heart disease; HF, heart failure; DBIL, direct bilirubin; AF, Atrial fibrillation; HT, heart transplantation; MD, mean difference; CR, Home-based CR; CBCR, center-based CR; AACVPR, American Association of Cardiovascular and Pulmonary Rehabilitation; AHA, American Heart Association; ACC, American College of Cardiology; RR, risk ratio; CI, confidence interval; NNT, number needed to treat; PCI, Percutaneous coronary intervention; RCT, Randomized controlled trial; BMI, Body Mass Index.
